# Effects of call reminders, short message services (SMS) reminders, and SMS immunization facts on childhood routine vaccination timing and completion in Ilorin, Nigeria

**DOI:** 10.4314/ahs.v21i2.57

**Published:** 2021-06

**Authors:** Rasheedat Ibraheem, Moshood Akintola, Mohammed Abdulkadir, Hafsat Ameen, Oladimeji Bolarinwa, Muhammed Adeboye

**Affiliations:** 1 University of Ilorin, Department of Paediatrics and Child Health; University of Ilorin Teaching Hospital, Paediatrics and Child Health; 2 Ladoke Akintola University of Technology Teaching Hospital, Department of Paediatrics; 3 University of Ilorin, Department of Epidemiology and Community Health

**Keywords:** Call reminders, short message services (SMS) reminders, SMS immunization, vaccination timing, Nigeria

## Abstract

**Background:**

Reminders via mobile devices deployed as short message services (SMS) or calls have been identified to be a useful strategy in improving routine immunization uptake in several countries.

**Objective:**

To identify the timeliness of appointments with reminders (calls or SMS), SMS health education and the routine care, and the vaccination completion rates in Ilorin, Nigeria.

**Method:**

Mother-infant pairs presenting for the first vaccination appointment were randomized into four (three interventions, one control) groups, each consisting of 140 participants. Intervention groups were reminders via calls (A), SMS reminders (B), immunization fact SMS messages (C) and controls on usual care (D). Reminders were made a day before the appointment while SMS immunization facts were sent at five weeks, nine weeks and eight months. Appropriate timing was defined as the scheduled visit ±3 days.

**Results:**

The immunization completion rates after the nine months' visit were 99.2%, 99.3%, 97% and 90.4% for Groups A, B, C and D respectively. Compared with controls, Group A had the highest odds [AOR 8.78 (6.10, 12.63)] of presenting at an appropriate time, followed by Group B [AOR 2.56 (1.96, 3.35)], then Group C [AOR 2.44 (1.87, 3.18)].

**Conclusion:**

Reminders/SMS immunization facts improve vaccination completion rates.

## Introduction

Routine vaccination against vaccine-preventable diseases (VPD) is a cost-effective intervention with a significant effect on reducing childhood morbidity and mortality. Despite the benefits of routine vaccination, the uptake and completion rates of vaccination remain low in the Africa Region.^1^ In Nigeria, the 2016–2017 Multiple Indicator Cluster Survey (MICS) identified the percentage of children aged 12–23 months who received all vaccinations recommended in the national immunization schedule by their first birthday was 23% while those that received pentavalent and measles vaccines were 33.0% and 42.0% respectively.^2^

Various studies have demonstrated a significant increase in drop-out between consecutive vaccines, with the drop-out being highest between pentavalent^3^ and measles vaccine.^3^, ^4^ A possible reason for this dropout is the longer interval between pentavalent and measles vaccine (three and a half months) compared to that between the earlier vaccines in the schedule (four weeks). With the long intervals, caregivers/mothers may forget, lose interest or fail to make out the time to keep the scheduled appointment for vaccination.^3^ Thus, scheduled reminders may prove important in ensuring caregivers take their infants for vaccines at the right time. Telephones provide a ready mechanism for delivering these reminders.

There has been an increase in the ownership and access to mobile devices in Nigeria in the last two decades.^5^ Most households have at least one mobile phone (identified as 74.4% in Nigeria from the 2016 MICS), and there has been a corresponding increase in the communication network coverage over the last few years.^2^ These mobile devices can be used to deliver information on the importance of vaccination as well as provide information on the vaccination schedules and appointments either as part of specific health education or reminders/recalls tailored to upcoming scheduled vaccinations for their infants. Reminders (by short messaging service or calls) have the potential of encouraging the caregiver to bring the child for vaccination as and when due, thus ensuring improved and timely vaccination uptake rates. Recent data from several countries support this potential in improving vaccination uptake.^6^–^13^ Text messages are simple and inexpensive, but the recipient needs to understand, synthesize and implement the message. This may be a challenge considering documented literacy rate among Nigerian women and men of 59.3% and 70.9% respectively.^2^ Indeed, a study about the preferences of mothers regarding reminders identified a majority preference for phone calls compared with text messages,^14^ with a higher preference for text message identified in mothers with postsecondary education.^14^, ^15^ In Africa, some studies have recommended the use of reminders as either SMS 6, 8, 12, 16 or calls,^10^ to improve vaccination coverage. There is however a dearth of studies that compared the effect of deployment of either calls or SMS for reminders with SMS health education about immunization facts on vaccination uptake. Thus, the general objective of the study was to identify the timeliness of appointments with reminders, (calls or SMS), SMS health education and routine care, as well as vaccination completion rates of infants brought for vaccination in Ilorin, Nigeria.

## Material and Method

This was a quasi-experimental study in which 140 subjects were assigned to one of four groups each. Three groups each had one intervention (SMS reminders, phone call reminders and SMS health education), while the last group in a different hospital had routine care. Subject recruitment occurred between August and December 2016. Each subject was followed until the time of the 5^th^ visit at 9 months of age and the study was completed in December 2017. The primary outcome of the study was the timing of receipt of each vaccine at each of the five visits, while the secondary outcome measure was vaccination completion rates. Patients in all groups received basic counselling about the importance of vaccination at the commencement of the study and during every visit.

### Study site

The study was conducted at two public hospital-based immunization centres in Ilorin (General Hospital, and Adewole Cottage Hospital). The hospitals, located in Ilorin West Local Government Area (LGA) of Kwara State, provide health care at the primary and secondary level within the State. One hospital was used for the intervention and the second hospital for the control.

The immunization centres provide vaccination to infants from Monday to Friday, except during public holidays. The vaccines supplied by the State Primary Health Care Development Agency (PHCDA) are administered to the infant at no cost to the parent. Services rendered at the immunization unit of the hospital include vaccination, growth monitoring, as well as nutrition and general health education. During the study, the vaccination schedule was recorded as-

- 1^st^ visit at birth- Bacille Calmette-Guerin (BCG), hepatitis B virus and oral polio vaccine (OPV-0).
- 2^nd^ visit at Six weeks: Pentavalent vaccine (Penta-1), OPV-1, and pneumococcal conjugate vaccine (PCV-1).
- 3^rd^ visit at Ten weeks: Penta-2, OPV-2, and PCV-2.
- 4^th^ visit at Fourteen weeks: Penta-3, OPV-3, PCV-3 and inactivated polio vaccine (IPV).
- 5^th^ visit at Nine months: measles and yellow fever vaccine

In the National Programme on Immunization (NPI) schedule, there is a scheduled visit for Vitamin A at six months but this visit was excluded from the study.

### Sample size determination

The primary outcome measure of the study was the proportion of children in each arm that had received immunization doses at an appropriate time. As no study had compared the interventions in this manner, a conservative estimate of 30% improvement in timeliness of vaccination doses was used for the interventions. With 80% power and level of significance α= 0.05, we estimated that 111 subjects needed to be recruited in each arm of the study. Considering the low completion rates for immunization in Nigeria 25.4% 17, we projected a 20% attrition rate for the study. Thus, a required sample size of 133 was calculated, which was rounded up to 140 in each arm.

### Inclusion/ exclusion criteria

Caregiver/Mother-infant pairs presenting for the first dose of vaccine in the five infant NPI schedule visits were eligible if they had a telephone contact number (either personal/ spouse/relative staying in the same house) and signed the consent to participate in the study. Mothers were excluded if they indicated they would not be present in the State for prolonged periods during the duration of the study.

### Ethical Consideration

The study was approved by the Kwara State Research Ethical Review Committee and written informed consent was obtained from the mother of each child.

### Subject screening, recruitment and enrolment

Adequate information about the study was given to mothers/caregivers, and thereafter informed consent obtained. Mother-infant pairs recruited from the facility used for the intervention were allocated by simple random sampling into either of the three intervention groups. The mother-infant pairs at the control facility were recruited consecutively. Recruitment was done by two research assistants who had received adequate training about the study objectives, and methodology. Registration details were obtained at recruitment and included demographic details of the mother/ caregiver, address, phone number of the mother (father if mother did not have a phone) and phone number of another contact that lives nearby the mother/ caregiver.

### Data collection

At baseline (1st visit), the participants' socio-demographic details, contact number, place of delivery and antenatal care during pregnancy were collected and recorded in the study proforma. At subsequent visits, the vaccination records were used to identify the children that presented at either of the study sites. When study duration elapsed, phone calls were made to all study participants who had not shown up for vaccination three months after the scheduled time. All those who could not be reached or followed-up were excluded from the analysis. Study participants who were contacted and confirmed they did not show up for vaccination, were considered not to have completed vaccination for each of the series of vaccinations.

### Intervention

The subjects and controls were in four groups, each consisting of 140 mothers/caregivers. Those recruited in the first group (A) received reminders via telephone calls, the second group (B) received reminders via text messages alone, a third group (C) received health education messages. All these groups had information on the timing and benefits of vaccinating children (routine health education) during each visit. The fourth group (D) received routine education alone. Reminders for vaccination appointments were sent a day before the appointed day for those in the first and second groups. Those in the third group were sent the health education message via SMS at three different intervals (five weeks, nine weeks and eight-month) between the collection of the first dose vaccine and the time of the fifth visit at nine months.

The MTN® network was used for all interventions.

### Phone call reminders

The reminders were given in the English language, +/- the local language of Yoruba. Attempts via phone call were continued until the phone call was picked. The number of the mother was repeatedly dialled three times and if no response, the father's number was called. Subsequent calls were alternated between the mother and the father in the evening till it was picked. The call message was, “Good day Sir/Ma, your child is due for the next vaccines tomorrow. Please bring your child for vaccination at the hospital at 8 am. Thank you.”

### SMS reminders

The SMS reminders were sent to either one or two contact numbers in instances where two numbers were provided (both parents). The SMS reminder sent was, “Dear parent, your child is due for the next vaccines tomorrow. Please bring your child for vaccination at the hospital at 8 am. Thank you.”

### SMS health education

The health education SMS immunization fact was, “Vaccination protects your child against diseases such as measles, yellow fever, tuberculosis, hepatitis B, poliomyelitis, pertussis, and tetanus. Your baby should receive the vaccines against these diseases at birth, six weeks, 10 weeks, 14 weeks and nine months at the nearest immunization unit. It is free.”

### Outcome measures

The outcome measures were the appropriateness of the timing of presentation/ receipt of vaccination dose for the five series of vaccinations scheduled, and the vaccination completion rates. An “appropriate timely appointment” was defined as the proportion of children vaccinated according to the NPI schedule within ±3 days of the scheduled appointment. Vaccinations before or after this interval were considered to be an “inappropriate timely appointment”. The general vaccination coverage was defined as the proportion of children who received all the vaccination for the five appointments. Appointments were defined as “missed” if patients did not come to receive the vaccine for which he/she was due. The immunization record book at each facility was used to check for presentation at each visit. For parents whose name could not be traced, call interviews were used to verify the immunization visit.

### Data analysis

Data were analyzed using the IBM® SPSS version 20.0 (IBM corporation, Virginia, U.S.A.) 2011 for Windows software package. The data collected on the proforma were transferred into a master sheet using numerical codes. Continuous variables were expressed as mean and standard deviation (SD), categorical variables as number and percentage. Timing of immunization was considered to be appropriate when it was received within ±3 days of the expected date. After the generation of frequency tables and simple proportions, the chisquare (χ2) and Student's t-tests were used to identify significant differences for categorical and continuous variables respectively. Logistic regression analysis was done to determine the predictors of vaccination completion at the fifth visit and the timeliness for each visit. Furthermore, the test of generalized equalizing equation (GEE) was used to create a model that identified predictors of presentation for each visit as well as the appropriateness of the time of presentation across the four groups. A p-value of 0.05 or less was considered statistically significant.

## Results

**Figure 1:**
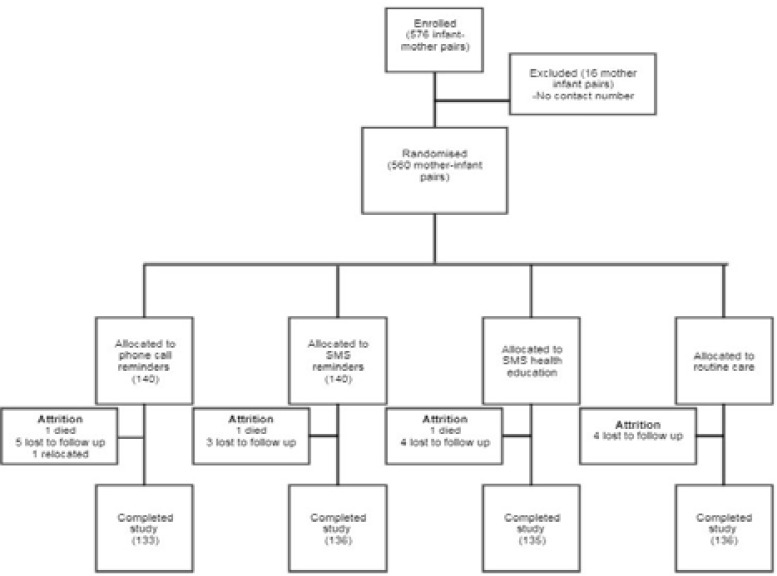
Flow chart of mother-infant pairs from enrolment to completion

Five hundred and seventy-six mother-infant pairs were recruited however 16 were excluded on account of both the mother and the father not having a mobile phone. Thus, 560 mother-infant pairs were available for randomization. One hundred and forty were allocated to each of the four arms of the study of phone call reminders (Group A); SMS reminders (Group B); SMS health information (Group C); and routine care (Group D). There were seven drop-outs from the study in the first group, while the remaining three groups had four, five and four drop-outs respectively. Thus, data from five hundred and forty mother-infant pairs was analyzed. This is summarized in Figure 1.

### Demographic characteristics of the study population

The mean (SD) age of the mothers was 28.7 (4.7) years, with 316 (58.5%) belonging to the 20–29years age group. Three hundred and seventy-three (66.6%) mothers practised Islam while the remaining 33.3% practised Christianity. Three hundred and forty (60.7%) mothers had attained post-secondary educational status.

A majority (510 mothers; 94.4%) of the mothers had antenatal care during the index pregnancy and 487 (90.2%) delivered in a hospital/ health centre. Two hundred and seventy-two (50.4%) of the infants were male and 203 (37.6%) were the first children of the mothers. Other characteristics of the mothers and infants are shown in Table 1.

**Table 1:** The demography of the study participants

Parameter	Frequency	Percentage
**Gender**		
Male	272	50.4
Female	268	49.6
**Place of delivery**		
Home/ traditional birth attendant place	33	6.1
Private hospital	68	12.6
Government hospital	419	77.6
Church	20	3.7
**Religion**		
Islam	373	69.1
Christianity	167	30.9
**Educational level of mother**		
None	1	.2
Primary	6	1.1
Secondary	193	35.7
Postsecondary	340	63.0
**Occupation of mother**		
Lecturer, Doctors, Large Business Owner	12	2.2
Nurses, Medium Business Owner,	93	17.2
Artisan	302	55.9
Petty Trader	16	3.0
Student, Subsistence Farmer	117	21.7
**Birth order**		
First	203	37.6
Second -Third	265	49.1
≥ Fourth	72	13.3

### Appropriateness of vaccination timing

The proportion of children who presented at an appropriate time decreased with each visit in each group (Table 2). The decrement was smallest among the call reminder groups and highest among the control group; 89.4%. 63.7%, 56.5% and 36.6% presented at an appropriate time for the nine-month visit in Group A, B, C and D respectively.

**Table 2:** Comparison of appropriateness of timing according to interventions/ nonintervention

Parameter	Intervention group			Routine care n (%)	Chi square	p-value

	Call reminder n (%)	SMS reminder n (%)	SMS immunization education n (%)			
**Six weeks' visit**						
Inappropriate	1(0.8)	4 (2.9)	2 (1.5)	60 (45.8)	175.643	<0.001
Appropriate	132(99.2)	132 (97.1)	133 (98.5)	71 (54.2)		
**10 weeks' visit**						
Inappropriate	9 (6.8)	27 (19.9)	18 (13.3)	36 (27.9)	22.935	<0.001
Appropriate	124(93.2)	109 (80.1)	117(86.7)	93(72.1)		
**14 weeks' visit**						
Inappropriate	17 (12.8)	41 (30.1)	47 (35.1)	42 (33.1)	20.637	<0.001
Appropriate	116 (87.2)	95 (69.9)	87(64.9)	85 (66.9)		
**9 months' visit**						
Inappropriate	14 (10.6)	49 (36.3)	57 (43.5)	78 (63.4)	77.167	<0.001
Appropriate	118 (89.4)	86(63.7)	74 (56.5)	45 (36.6)		

Table 3 shows that at 14 weeks, only Group A presented within an appropriate time when compared with controls, OR 3.37, 95%C.I.=1.80–6.33. Compared with controls, the odds of presenting at an appropriate time was 14x, 3x and 2x for Groups A, B and C respectively at the nine months' vaccination appointment.

**Table 3:** Logistic regression showing appropriateness of timing at each visit according to intervention compared with the non-intervention group

Parameter	B	S.E.	Sig.	OR (95%C.I.)
**Six weeks' visit**				
Calls	4.714	1.019	<0.001	111.55(15.14–821.92)
SMS	3.328	0.537	<0.001	27.89(9.74–79.89)
SMS health education	4.029	0.734	<0.001	56.20(13.34–236.71)
None				
**10 weeks' visit**				
calls	1.674	0.397	<0.001	5.33(2.45–11.62)
SMS	0.446	0.291	0.125	1.56(0.88–2.77)
SMS health education	0.923	0.320	0.004	2.52(1.34–4.71)
None				
**14 weeks' visit**				
Calls	1.215	0.321	<0.001	3.37(1.80–6.33)
SMS	0.135	0.266	0.610	1.15(0.68–1.93)
SMS health education	-0.089	0.261	0.733	0.92(0.55–1.53)
None				
**Nine months' visit**				
Calls	2.682	0.339	<0.001	14.61(7.52–28.39)
SMS	1.113	0.259	<0.001	3.04(1.83–5.05)
SMS health education	0.811	0.257	0.002	2.25(1.36–3.73)
None				

The GEE test results of the combined within and between subjects of the effects of the interventions is shown in Table 4. Mother-infant pairs in each of the three intervention arms had higher likelihoods of presenting for subsequent immunization visits than those in the control (usual care) group. The SMS reminder group had the highest odds [AOR 36.20 (CI 4.94, 265.42)] of presentation for the immunization compared to those in Group D. Also, the three intervention arms had a higher likelihood of presenting for vaccination visits at the appropriate time than the control (usual care) groups.

**Table 4:** Generalized Estimating Equation (GEE) showing effects of intervention arms on presentations and appropriate timing for subsequent immunization

Variable	Beta Co-efficient	S. E.	Wald χ^2^	Adjusted Odds (95% CI)	p-value
**Presentation for immunization**					
Intercept	2.71	0.18	233.76	15.00 (10.60–21.23)	**<0.001**
Call reminders	3.57	1.02	12.31	35.40 (4.83–259.56)	**<0.001**
SMS reminder	3.59	1.02	12.47	36.20 (4.94 – 265.42)	**<0.001**
SMS immunization facts	1.97	0.48	16.55	7.133 (2.77–18.38)	**<0.001**
Routine care (reference category)	-	-	-	-	-
**Appropriate timing**					
Intercept	0.31	0.09	11.84	1.36 (1.14 – 1.62)	**0.001**
Call reminders	2.17	0.19	136.96	8.78 (6.10 – 12.63)	**<0.001**
SMS reminder	0.94	0.14	47.43	2.56 (1.96 – 3.35)	**<0.001**
SMS immunization facts	0.89	0.14	42.75	2.44 (1.87 – 3.18)	**<0.001**
Routine care (reference category)	-	-	-	-	-

S.E. = Standard error, χ^2^ = Chi square test, * = significant, AOR = Adjusted Odd Ratio.

The call reminder group had the highest odds [AOR 8.78 (6.10, 12.63)] of presenting at an appropriate time for the vaccination compared to those in group 4 (control group). This is followed by the SMS reminder group [AOR 2.56 (1.96, 3.35)] and then the SMS immunization facts group [AOR 2.44 (1.87, 3.18)].

### Vaccination co mpletion rates

Overall, 535 (99.1%), 533 (98.7%), 530 (98.1%) and 521 (96.5%) children received vaccinations at the 2nd, 3rd, 4th and 5th visits respectively.

**Figure 2:**
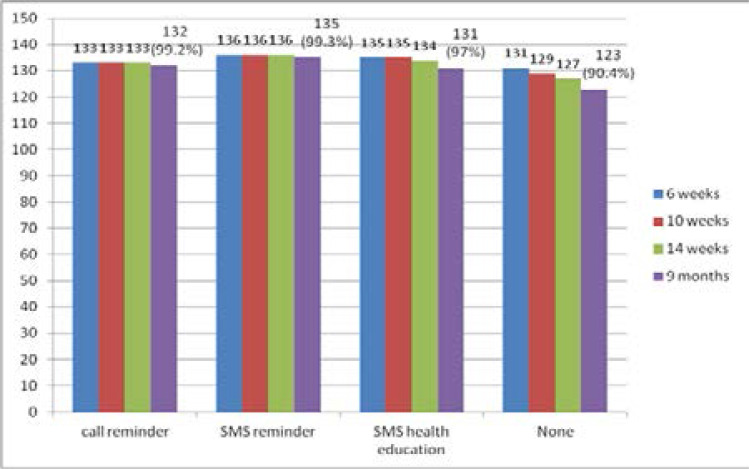
The proportion of children who presented for each visit

The immunization completion rates at nine months were 99.2%, 99.3%, 97.0% and 90.4% for Groups A, B. C and D respectively. The vaccination completion rates for the earlier visits are as shown in Figure 2.

## Discussion

Phone call reminders were associated with the highest appropriateness of presentation timing for vaccinations in the current study. This finding associated with call reminders is similar to reports of studies in the South-Western and South-Eastern regions of the country,^10^, ^18^, ^19^ and an earlier systematic review.^20^ The studies in Ibadan and Abakaliki in Nigeria were with real-time phone call reminders like the current study while the Akure study (also in Nigeria) used automated phone calls. Advantages of real-time phone call reminders include callbacks following non-response and response to possible questions and clarifications during calls. However, this would be more expensive than the automated call reminders as more personnel are required for the former.

The SMS reminders improved the proportion of children that received the ninth month vaccines. However, the proportion of children that presented at an appropriate time for the various visits in the SMS reminder declined by more than 10% for each visit compared to the call reminder groups that declined by about 5%. These SMS reminder findings on the improvement of routine vaccination had earlier been reported in a study in the South-South region of Nigeria.^12^ However, the earlier study had also identified that those who received SMS reminders were 1.5 times more likely to present early for the 4^th^ vaccination which was not identified in the current study. This may be due to a difference in study definitions which was classified as ‘delayed’ after a four-week interval post-scheduled date in the earlier study as opposed to the current study definition of ‘inappropriate’. The proportion of children from the SMS reminder group who received 3^rd^ visit vaccinations at an appropriate time in this study was 69% which is slightly lower than the 82% reported from Zimbabwe.^6^

The SMS immunization facts improved uptake of routine vaccination compared to the non-intervention group, though, the proportion was lower than the reminder groups. Furthermore, for the fifth visit at 9 months, those with SMS immunization facts were twice as likely to present at an appropriate time compared to the control group. The implication is that repeated messages on immunization facts sent to caregivers may likely have an impact on the uptake and timeliness of routine immunization. Health education via mobile technology has been shown to improve child care services.^21^, ^22^ Advantages of the SMS immunization facts include educating the caregiver about the disease conditions that vaccination protects against and stating the timing of vaccination among others. The messages remain on the phone and are available for viewing at any time serving as not just an educational tool but also a reminder on the importance of vaccination. In Nigeria, the use of mobile application SMS messages and reminders is already being deployed to provide antenatal care counselling as well as uptake in some States.^23^ A similar strategy can be deployed to improve immunization uptake and timing.

## Conclusion

Phone call reminders, SMS reminders and SMS education improved the immunization completion rates in the study area. However, call intervention was associated with the highest proportion of completion rates and appropriateness of presentation timing for vaccinations. Deployment of mobile phone reminders/immunization facts would be a useful strategy to improve not only immunization uptake but also its completion rates in Nigeria.

## Limitations

The study has some limitations including the fact that the respondents were from a single local government area with an urban setting in the state and thus the result cannot be generalized to either rural or semi-urban areas. Also, the study excluded caregivers without contact phone which introduced a selection bias at the onset of the study. This category of caregivers is likely to default on vaccination, therefore a possible consideration for future study could involve utilizing the contact of a neighbor/ relative for the reminder. Furthermore, while the service standards at both centres are similar, a baseline on the timeliness of presentation for vaccination appointments as well as completion rates was not identified. This may also have some effect on the interpretation of the results.
